# Serum IRAP, a Novel Direct Biomarker of Prediabetes and Type 2 Diabetes?

**DOI:** 10.3389/fmolb.2020.596141

**Published:** 2021-02-16

**Authors:** Candice Trocmé, Nicolas Gonnet, Margaux Di Tommaso, Hanen Samouda, Jean-Luc Cracowski, Claire Cracowski, Stéphanie Lambert-Porcheron, Martine Laville, Estelle Nobécourt, Chiraz Gaddhab, Allan Le Lay, Torsten Bohn, Christine Poitou, Karine Clément, Fahd Al-Mulla, Milad S. Bitar, Serge P. Bottari

**Affiliations:** ^1^Department of Biochemistry, Molecular Biology and Environmental Toxicology, Centre Hospitalier Grenoble-Alpes, La Tronche, France; ^2^Centre d'Investigation Clinique, Centre Hospitalier Grenoble-Alpes, La Tronche, France; ^3^Population Health Department, Nutrition and Health Research Group, Luxembourg Institute of Health, Luxembourg, Luxembourg; ^4^Medical School, Université Grenoble Alpes, La Tronche, France; ^5^INSERM U1042 Laboratoire Hypoxie et Physiopathologies cardiovasculaires et respiratoires (HP2), Grenoble, France; ^6^Centre de Recherche en Nutrition Humaine Rhône-Alpes, Pierre-Bénite, France; ^7^CH Lyon Sud, Lyon, France; ^8^INSERM U1060 Laboratoire de Recherche en Cardiovasculaire, Métabolisme, diabétologie et Nutrition, Oullins, France; ^9^Department of Endocrinology, Metabolic Diseases and Nutrition, Centre Hospitalier Universitaire de La Réunion, Saint-Denis, France; ^10^Department of Pediatrics, Diabetes and Endocrinology Care, Centre Hospitalier de Luxembourg, Luxembourg, Luxembourg; ^11^CHU Grenoble-Alpes, Department of Biochemistry, Molecular Biology and Environmental Toxicology, Grenoble, France; ^12^INSERM UMR-S 1269, NutriOmics, Paris, France; ^13^Medical School, Sorbonne Universités, Paris, France; ^14^Department of Genomics and Bioinformatics, Dasman Diabetes Institute, Kuwait City, Kuwait; ^15^Department of Pharmacology, Faculty of Medicine, Kuwait University, Kuwait City, Kuwait; ^16^GREPI, UMR5525 Techniques de l'Ingénierie Médicale et de la Complexité Informatique, Mathématiques et Applications, Grenoble (TIMC-IMAG), La Tronche, France; ^17^Faculté de Médecine, Université Grenoble Alpes, La Tronche, France; ^18^Centre Hospitalier Grenoble-Alpes, La Tronche, France

**Keywords:** IRAP, diabetes, biomarker, prediabetes, diagnosis, screening, GLUT4

## Abstract

Insulin resistance (IR), currently called prediabetes (PD), affects more than half of the adult population worldwide. Type 2 diabetes (T2D), which often follows in the absence of treatment, affects more than 475 million people and represents 10–20% of the health budget in industrialized countries. A preventive public health policy is urgently needed in order to stop this constantly progressing epidemic. Indeed, early management of prediabetes does not only strongly reduce its evolution toward T2D but also strongly reduces the appearance of cardiovascular comorbidity as well as that of associated cancers. There is however currently no simple and reliable test available for the diagnosis or screening of prediabetes and it is generally estimated that 20–60% of diabetics are not diagnosed. We therefore developed an ELISA for the quantitative determination of serum Insulin-Regulated AminoPeptidase (IRAP). IRAP is associated with and translocated in a stoechiometric fashion to the plasma membrane together with GLUT4 in response to insulin in skeletal muscle and adipose tissue which are the two major glucose storage sites. Its extracellular domain (IRAPs) is subsequently cleaved and secreted in the blood stream. In T2D, IRAP translocation in response to insulin is strongly decreased. Our patented sandwich ELISA is highly sensitive (≥10.000-fold “normal” fasting concentrations) and specific, robust and very cost-effective. Dispersion of fasting plasma concentration values in a healthy population is very low (101.4 ± 15.9 μg/ml) as compared to those of insulin (21–181 pmol/l) and C-peptide (0.4–1.7 nmol/l). Results of pilot studies indicate a clear correlation between IRAPs levels and insulin sensitivity. We therefore think that plasma IRAPs may be a direct marker of insulin sensitivity and that the quantitative determination of its plasma levels should allow large-scale screening of populations at risk for PD and T2D, thereby allow the enforcement of a preventive health policy aiming at efficiently reducing this epidemic.

## State of the Art and Current Issues

Type 2 diabetes (T2D) is a severe chronic disease whose incidence has been increasing continuously over the past decades becoming a major public health and socio-economic issue (Herman and Zimmet, 2012; Menke et al., 2015; International Diabetes Federation, 2019). T2D does not develop abruptly, but is preceded by a gradually worsening impaired glucose tolerance or insulin resistance, now generally referred to as prediabetes (Perreault, 2000; Edwards and Cusi, 2016). This disease which is essentially asymptomatic, is due to the progressive appearance of a resistance of target tissues to the metabolic actions of insulin (Kahn, 1996). One of the most extensively characterized initial signs of prediabetes is a reduction of the rate of glucose uptake by the insulin responsive cells after a meal (Garvey et al., 1998; Jung and Lee, 1999). This together with increased hepatic gluconeogenesis leads to higher levels of postprandial glycemia and to a delayed return to basal glycemia levels. These two phenomena are usually accompanied by increased postprandial insulin levels. Additional features are usually hypertriglyceridemia, mild hypertension and overweight.

The situation generally worsens with time over a period which can take several years, to finally result in fasting hyperglycemia which is the definition of type 2 diabetes (Geiss et al., 2010).

Contrary to type 1 diabetes which is due to a severe decrease in insulin secretion by the pancreas, type 2 diabetes is due to defects in signal transduction between the insulin receptor and the molecular mechanisms involved in glucose uptake and other metabolic actions of insulin. These defects, make the cells resistant to the metabolic actions of insulin (Kahn, 1992, 1996). Whereas, not all tissues, e.g., the brain and the liver, depend on insulin for glucose uptake, the major glucose storage tissues, i.e., skeletal muscle and adipose tissue do. In addition, insulin is no longer able to inhibit gluconeogenesis, especially by the liver, thereby contributing to the progressive worsening of hyperglycemia. Numerous studies have shown that in the absence of early and adequate management of insulin-resistance, prediabetes almost invariably evolves to T2D (Perreault, 2000; Herman and Zimmet, 2012; Kerrison et al., 2017; Braga et al., 2019). Currently the International Diabetes Foundation (IDF) estimates that more than 460 million people have diabetes worldwide. Seventy five percent of the individuals having diabetes (> 350 million) are aged between 20 and 64 years and are therefore part of the working age population which explains the huge socio-economic burden of the disease (International Diabetes Federation, 2019).

T2D is the first cause of death in adults worldwide, namely about 4.2 million people, i.e., 11.3% of deaths. It is also a major cause of disability (Gregg et al., 2016; International Diabetes Federation, 2019).

It is now widely recognized that prediabetes carries an equal risk of morbi-mortality as T2D (Huang et al., 2016; Casagrande et al., 2018b) with regard to cardiovascular diseases (Balakumar et al., 2016; Strain et al., 2018) and/or cancer (Scappaticcio et al., 2017). Current IDF estimations emphasize that more than 375 million people worldwide have prediabetes (Bullard et al., 2018; International Diabetes Federation, 2019). The total number of people living with a high risk of developing insulin-resistance associated diseases, essentially cardiovascular diseases and cancer, is therefore ≥ 835 million individuals. Moreover, the IDF considers that approximately half of the population having diabetes is undiagnosed and therefore unaware of having the disease (International Diabetes Federation, 2019).

Finally, about 16% of the pregnant women, i.e., more than 20 million women worldwide, develop diabetes during pregnancy. This gestational diabetes often results in potentially severe complications either during pregnancy or at the parturition and even later on in life both for the infant and the mother (Chiefari et al., 2017; Casagrande et al., 2018a; Plows et al., 2018).

From an economic point of view, the IDF estimated last year's worldwide direct cost of diabetes at 760 billion USD and its indirect costs at 455 billion USD (in 2015) (International Diabetes Federation, 2019).

Despite these impressive figures and the grim projections of the progression of the prevalence of these diseases for the next decades, there is so far no simple, reliable, and cost-effective diagnostic test for T2D and even less so for prediabetes (Muniyappa et al., 2008; Malkani and DeSilva, 2012; Wolffenbuttel et al., 2013; Xiang et al., 2014; Gloyn and Drucker, 2018).

Currently, all diagnostic tests for T2D are based either on the determination of glycemia, insulin or its secreted precursor C-peptide levels or on the assessment of the consequences of hyperglycemia, e.g., hemoglobin glycation (HbA1c levels). The most common screening test used is random plasma glucose testing (RPG). In an elegant paper, Herman's group showed that this method results in an extremely high overestimation of the incidence of T2D using a cut-off value of 130 mg/dl (Johnson et al., 2005). In order to try to standardize screening it has been recommended to assay fasting plasma glucose levels instead. It turned out that using a cut-off value of 126 mg/dl, the sensitivity of FPG ranges between 35 and 59% and the specificity between 85 and 95%, comparable to the sensitivity and specificity of an RPG cut-off point of 160 mg/dl (Blunt et al., 1991; Bortheiry et al., 1994; Engelgau et al., 2000). Another, more logical approach for the screening of T2D considering that it is due to insulin resistance, is to measure post-prandial glycemia (PPG). The major drawback of this method is the considerable inter-individual and intra-individual variability of PPG (Venn and Green, 2007; Rohling et al., 2019) due to high interpersonal variability in post-prandial glycemic responses to the same food (Zeevi et al., 2015). It is thus obvious now that these tests cannot be used for screening for T2D and even less so for PD (Bansal, 2015).

Another parameter whose measurement has been proposed for screening of T2D, is HbA1c. The rationale behind it is that HbA1c should reflect an integrated measurement of glycemia over a period of ~3 months as compared to the real time punctual information represented by FPG and PPG. Unfortunately, HbA1c levels are also strongly determined by genetic factors (Cohen et al., 2006; Bloomgarden et al., 2008; Cavagnolli et al., 2017) and do therefore show important interindividual variability independently of glycemia. In agreement with this, a lack of correlation with FPG and PPG have been consistently reported (Bonora et al., 2001; E. van 't et al., 2010; Cavagnolli et al., 2011; Nathan et al., 2014), as well as other discrepancies (Gomez-Perez et al., 1998; Bonora and Tuomilehto, 2011; Tucker, 2020).

Therefore, whereas it is a useful marker for the follow-up of glycemia in T2D patients, each patient acting as his own control, it obviously cannot be used as a diagnostic neither as a screening assay (Gomez-Perez, 2015).

Considering the lack of sensitivity and specificity of these static markers, dynamic tests have been and are currently being used. The most widely used is the oral glucose tolerance test (OGTT) first described by JW Conn in 1940, in which patients are challenged with glucose after which their glycemia and insulinemia are monitored for 2 h or more (Matthews et al., 1985; American Diabetes Association, 2020). Based on the glycemia and insulinemia values during the 2-h duration of OGTT, Matsuda has proposed an equation which fits better with the data obtained using the euglycemic hyperinsulinemic clamp, considered to be the gold standard (Matsuda and DeFronzo, 1999). This index has further been improved using the AUCs for both parameters (Abdul-Ghani et al., 2007). Nevertheless, the intrinsic problem of this dynamic test is the important intra- and interindividual variability of glycemia and insulinemia, even if this issue is somewhat alleviated by integrating the values measured at several time points. Thus, whereas OGTT may represent an improvement for the diagnosis of T2D, the immobilization of the patients for more than 2 h it requires, precludes it from being used for screening purposes. This is even more so for the euglycemic hyperinsulinemic clamp which can only be used in research settings.

In an attempt to find a solution to this issue, an index based on the mathematical modeling of the steady-state basal plasma glucose and insulin concentrations feedback loop has been developed. It has been proposed by the authors that comparison of a patient's fasting values with the model's predictions allows a quantitative assessment of the contributions of insulin resistance and deficient beta-cell function to the fasting hyperglycaemia (homeostasis model assessment, HOMA) (Matthews et al., 1985). However, as indicated by the authors, “The low precision of the estimates from the model (coefficients of variation: 31% for insulin resistance and 32% for beta-cell deficit) limits its use (Matthews et al., 1985).” Most important are, as stated by the authors, the facts that 1° The HOMA model is a model of the glucose-insulin feedback system in the homeostatic (overnight-fasted) state and 2° it has been designed to predict pancreatic β-cell function (%β) and insulin sensitivity (IS) in the fasting steady state. Its initial aim was to provide an accurate representation of physiology and successfully predict the homeostatic responses to an intravenous glucose infusion (Levy et al., 1998).

This model has been extrapolated to a clinical use to determine IS and %β and from there to diagnose diabetes despite the caveats and inappropriate uses pointed out by the authors (Wallace et al., 2004). Obviously, the value of the HOMA index essentially depends on the quality of the sample measurements (Matthews et al., 1985) which brings us back to the issue of FPG with its high degree of intra-and interindividual variability which also holds for insulinemia.

Many other indices have been proposed based on direct, i.e., insulin infusion-based tests, and indirect, i.e., insulinemia measurements, methods. The advantages and caveats of the major indices for clinical purposes has been reviewed in detail by Matsuda in 2010 (Matsuda, 2010). The correlations of 35 indices with IS and 1/IS obtained from IVGTT and standard hyperglycemic breakfast test have been reported and are indicative of the important discrepancies between most of them (Aloulou et al., 2006). More interesting even is the comparison of several of these indices in similar settings between diabetics and healthy subjects (Brun et al., 2013). The data reported unambiguously show that whereas there is a good correlation of some indices in healthy subjects, this is not the case in patients with diabetes. This thus once more raises the issue of extrapolating indices which reflect physiological glucose homeostasis to clinical settings.

In summary, all available methods used so far for the diagnosis of T2D suffer from more or less serious drawbacks and particularly the tests based on single time point determinations. This is obviously even more true for PD where increases in glycemia and insulinemia are less pronounced than in T2D.

The most reliable diagnostic test being OGTT based, there is thus as yet no reliable method available allowing for the screening of T2D and PD.

Recent work aimed at finding novel biomarkers of PD and T2D has resulted in the identification of copeptin, which is the C-terminal fragment of pre-provasopressin (Enhorning et al., 2011; Wannamethee et al., 2015; Roussel et al., 2016; Jensen et al., 2019). The mechanisms which link the secretion of copeptin to insulin-resistance are however still unclear. It is possible that increased copeptin levels in T2D are due to decreased translocation of IRAP which has been reported to degrade vasopressin, thereby triggering the secretion of its precursor pre-pro-vasopressin. Interestingly however copeptin appears to be an interesting independent marker of renal and cardiovascular complications in T2D (Riphagen et al., 2013; Velho et al., 2013; Bar-Shalom et al., 2014; Zhu et al., 2016; Potier et al., 2019).

## A Novel Diagnostic Approach

Our approach has therefore been to shift from an integrative parameter, glycemia, to a specific and exclusively insulin-dependent parameter, glucose uptake. Indeed, insulin resistance which is the defect underlying prediabetes, T2D and gestational diabetes, always involves altered insulin-mediated glucose uptake (Kahn, 1996).

The mechanism through which insulin stimulates cellular glucose uptake, is by inducing the translocation of the vesicles which contain the glucose transporter GLUT4, from the cytoplasm to the plasma membrane (Bryant et al., 2002). As a result, these vesicles fuse with the plasma membrane and the GLUT4 proteins become inserted there. Since there is an important concentration gradient of glucose from the extracellular fluid to the cytosol, glucose will enter the cell by passive diffusion and will be instantaneously phosphorylated to enter the metabolic pathways. Thus, cytosolic glucose concentrations always remain extremely low, maintaining the concentration gradient.

Apart from this pathway, there is no other additional or alternative way for glucose to enter cells in an insulin-dependent fashion. Certain cells and organs like the brain and the liver do not rely on insulin for glucose uptake and express other glucose transporter isoforms which are located constitutively in the plasma membrane.

The major issue with this approach was to find the appropriate biomarker. Indeed, determining plasma membrane vs. vesicular GLUT4, is not only complex but also invasive as it requires muscle or adipose tissue biopsies. Such an approach is obviously not suitable for routine and large-scale screening or diagnosis of insulin resistance.

The challenge was thus to find a plasmatic or serum biomarker of GLUT4 vesicle translocation.

GLUT4 vesicles contain a series of other proteins, most of which are essentially involved in the translocation process (Bogan and Kandror, 2010).

**Figure 1:**
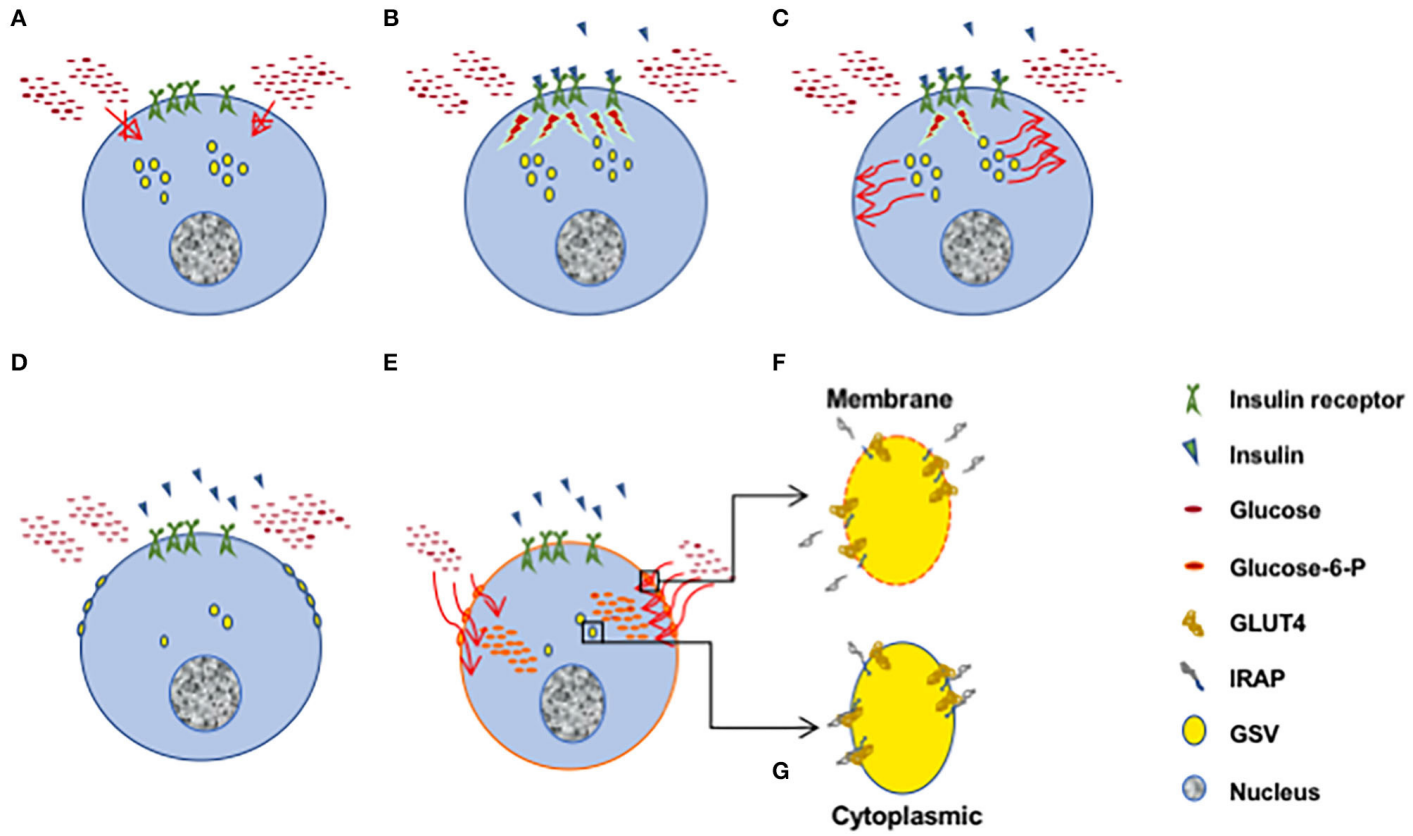
Insulin-mediated glucose uptake through GLUT4. **(A)** In the absence of insulin, glucose cannot enter the cell. **(B)** Secreted insulin binds to its receptors and triggers multiple signaling pathways. **(C,D)** Among these pathways, insulin triggers the translocation of part of the GLUT4 containing vesicles (GSV) to the plasma membrane. **(E)** Upon fusion with the plasma membrane (PM), GLUT4 molecules are inserted in the PM and allow glucose to enter the cell. Glucose is rapidly phosphorylated to glucose-6-phosphate (G-6-P) as an intermediate metabolite in glycolysis, pentose pathway and glycogen synthesis. **(F)** In the fused GSVs, IRAP is inserted the PM together with GLUT4 and its C-terminal extracellular domain is cleaved and secreted in the bloodstream. Subsequently, GSV proteins will be recycled and incorporated in newly formed cytoplasmic GSVs. **(G)** Part of the GSVs stay in the cytoplasm and are available for future translocation. IRAP molecules are intact.

Interestingly, among these vesicular proteins, there is an aminopeptidase which appears to be closely associated with GLUT4 in a stoechiometric fashion. A peculiarity of this protein called Insulin-Regulated AminoPeptidase (IRAP) which was identified and cloned in 1994 (Mastick et al., 1994; Keller et al., 1995; Ross et al., 1996), is that its large 160 kDa extracellular domain is cleaved by metalloproteases, ADAM 9 and 12 and thus secreted in the bloodstream (Figure 1) (Ito et al., 2004), similar to angiotensin-converting enzyme (ACE) (Costerousse et al., 1992).

Since insulin triggers the translocation of the GLUT4 vesicles to the plasma membrane, the circulating levels of IRAP should reflect the amount of IRAP and GLUT4 which are translocated to the plasma membrane (Figure 1)and hence reflect the degree of insulin sensitivity.

In agreement with this hypothesis, data from the literature indicate a decrease of IRAP translocation in response to insulin in adipocytes and skeletal muscle in diabetic rats (Takeuchi et al., 2006) and in patients with type 2 diabetes (Garvey et al., 1998; Maianu et al., 2001). It is therefore reasonable to assume that circulating IRAP (IRAPs) levels will be decreased in people having prediabetes or diabetes.

IRAPs may therefore be a direct marker of insulin response and sensitivity. Accordingly, its quantitative determination by a simple and robust method could allow the screening of populations at risk for prediabetes, type 2 diabetes and gestational diabetes.

Similar to what had been done previously by other investigators for measuring circulating angiotensin-converting enzyme (ACE) (Danilov et al., 1996), we attempted to develop an assay for the quantitative determination of IRAPs in serum.

**Figure 2:**
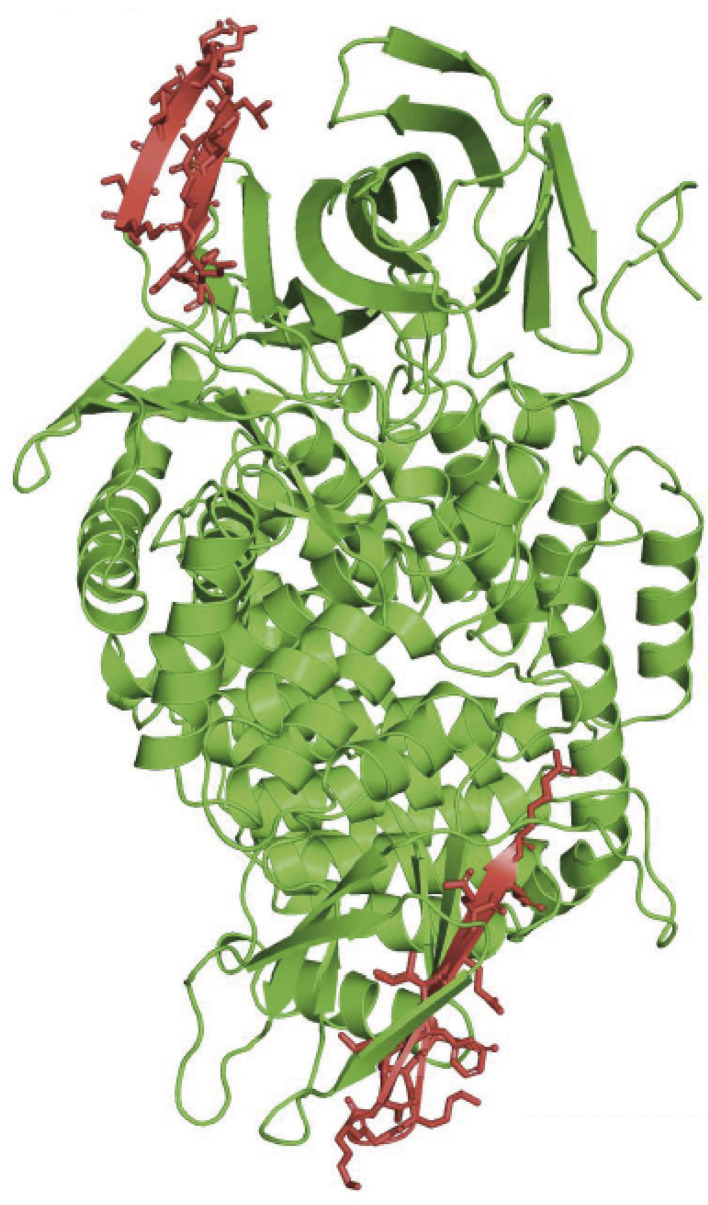
Structure of the cleaved extracellular domain of IRAP: 3-dimensional model of the cleaved extracellular domain of IRAP. Epitopes recognized by the monoclonal antibodies used in the ELISA are in red.

After having investigated and tried different techniques, we chose to further develop a sandwich ELISA using two monoclonal antibodies directed against two epitopes of the globular extracellular domain of IRAP (Figure 2) identified by 3-D modeling (Mpakali et al., 2015). The ELISA is calibrated with recombinant human IRAPs obtained through (over)expression in mammalian cells.

The assay is highly specific for IRAP and does not display any cross-reactivity with other related aminopeptidases under the analytical conditions. Its sensitivity is 10 ng/ml for reference values around 100 μg/ml in healthy volunteers under fasting conditions. This assay and the diagnostic applications of IRAP have been patented under the reference PCT/FR2009/05133 and published as WO 2010/001079.

**Figure 3:**
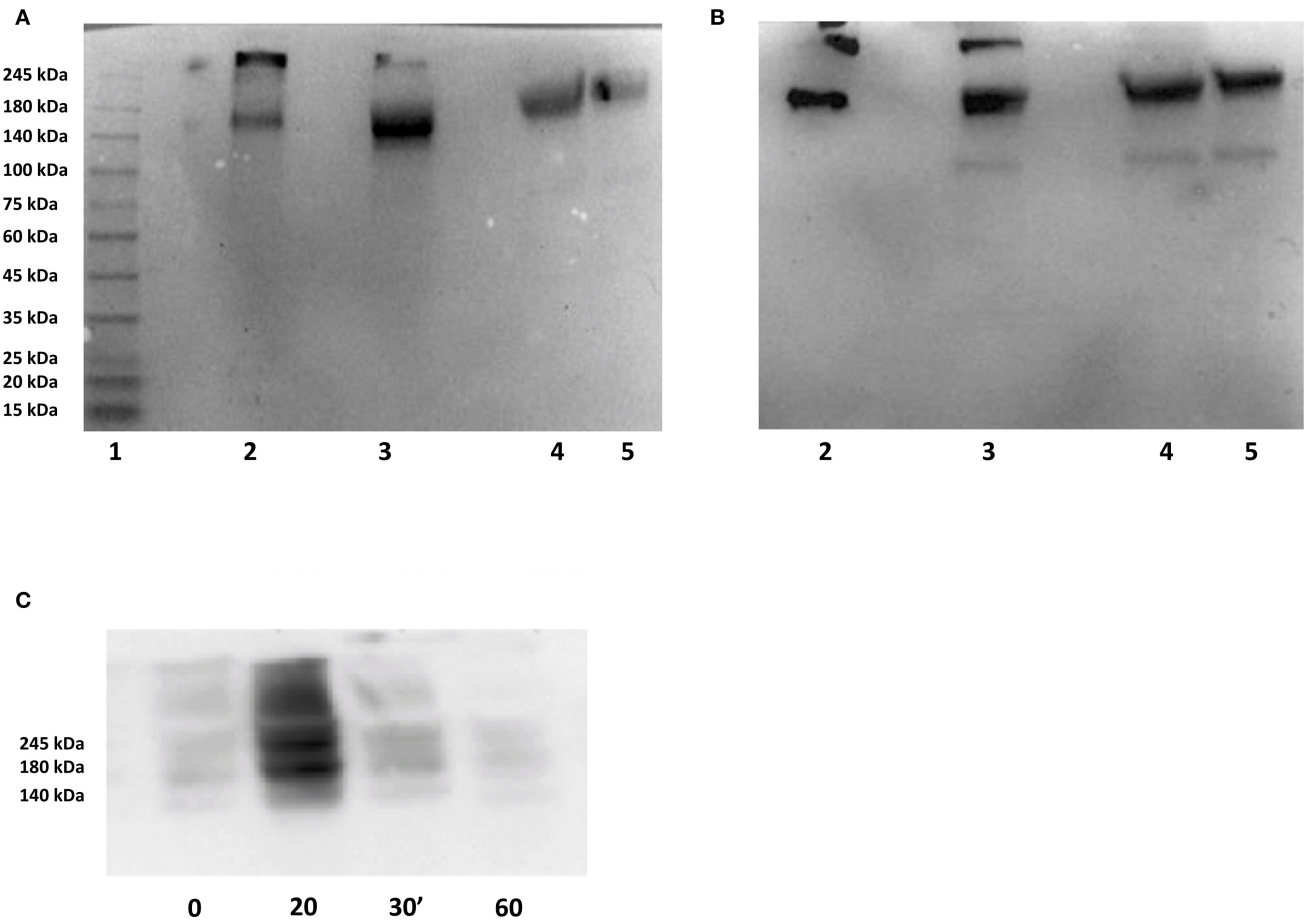
Immunoblots of the secreted domain of IRAP. **(A,B)** Samples were submitted to SDS PAGE on 4–15% gradient gels under non-reducing conditions and transferred to PVDF membranes by semidry blotting. Blots were probed resp. with 4G6 mAb **(A)** and 40C10 mAb **(B)** and revealed using an anti-mouse-HRP antibody (preadsorbed to human IgG) and luminol. Lanes: 1: molecular weight markers, 2: -, 3: 150 ng recombinant human IRAP secreted domain (rhuIRAP sd), 4: -, 5: 1.5 μL human serum spiked with 150 ng rhuIRAPsd, 6: 1,5 μL fetal bovine serum, 7: 1.5 μL human serum, 8: 0.75 μL human serum. **(C)** Sera obtained during an insulin-induced hypoglycemia test were submitted to native PAGE on a 4–15% gradient gel under non-reducing conditions and transferred to PVDF membranes by semidry blotting. The blot was probed with 4G6 mAb and revealed using an anti-mouse-HRP antibody (preadsorbed to human IgG) and luminol. Indicated times are in minutes following insulin injection.

**Figure 4:**
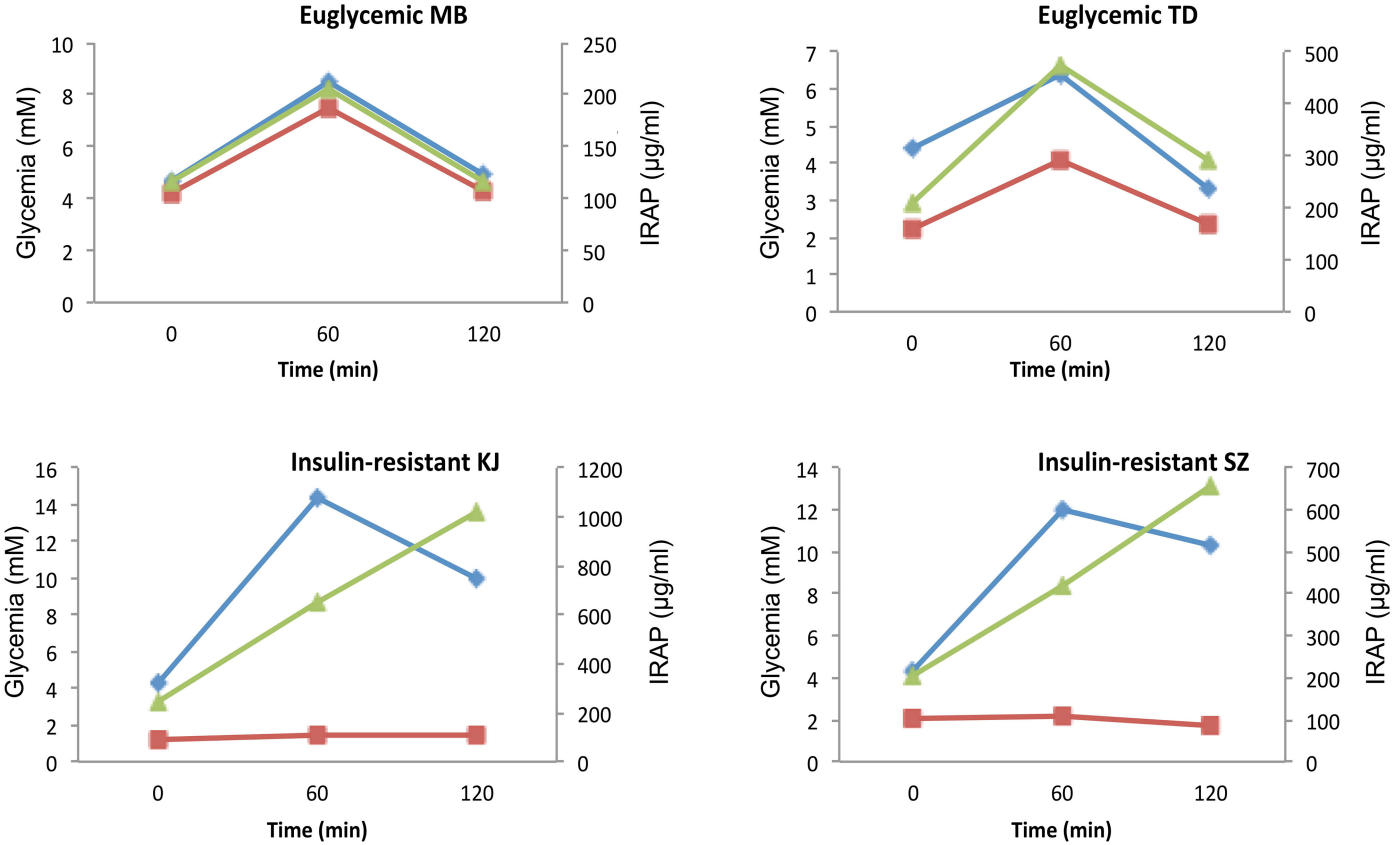
Glycemic, insulinemic, and serum IRAP concentration profiles during OGTT. Representative three-time points OGTT profiles in two euglycemic (MB and TD) and two severely insulin-resistant diabetic (KJ and SZ) patients. Glycemia, blue; insulinemia, green; IRAP, red.

Clinical trials to validate the diagnostic interest of this assay are currently under way. Preliminary data from pilot studies indicated that IRAPs is cleared from the circulation within 1 hr and that (Figure 3), in healthy individuals, its levels appear to follow glycemia and insulinemia (Figure 4). Interestingly, the dispersion of the IRAP concentrations determined in healthy volunteers under fasting conditions using this assay is low (101.4 ± 15.9 μg/ml) as compared to glycemia, insulinemia (21–181 pmol/l) or C-peptide (0.4–1.7 nmol/l), adding to its potential clinical value as a biomarker.

## Preliminary Results

As shown in Figures 3A,B, immunolabeling of serum IRAP after SDS-PAGE with either of both monoclonal antibodies used in the ELISA, i.e., 4G6 for capture and 40C10 for detection, reveals a major band with an apparent molecular weight (M_r_) of approximately 160 kDa. Another band with a higher M_r_ between 300 and 400 kDa seen in the recombinant human IRAP secreted domain preparation probably corresponds to dimerization. Conversely, bovine serum does not show any detectable IRAP, in agreement with the absence of a proteolytic cleavage site required for its shedding. Interestingly, the mAb 40C10 also detects a minor band with an M_r_ of ~100 kDA which may correspond to a proteolytic fragment.

Figure 3C shows that in serum samples obtained during an insulin-induced hypoglycemia test, IRAP reaches a peak concentration within 20 min after insulin injection and is cleared within 60 min. The mechanisms involved in IRAP clearance are not elucidated yet but probably involves proteolysis as we observed a proteolytic fragment with one of the monoclonal antibodies (Figure 3B). The PAGE performed under “native” conditions shown in this figure also indicates that IRAP forms oligomers. These dissociate in the presence of SDS, yielding a single band with an apparent molecular weight of approximately 160 kDa as shown in Figures 3A,B. Effective dissociation of the oligomers is obviously a key feature of the ELISA.

As expected, IRAP levels do not follow insulinemia in severe insulin resistance. Figure 4 shows typical glycemia, insulinemia and serum IRAP concentration profiles during OGTT in two euglycemic and two severely insulin-resistant diabetic patients. Whereas, serum IRAP levels correlate with glycemia and insulinemia in the two euglycemic patients, IRAP levels do not increase in response to increased insulinemia be it at 60 or 120 min after glucose intake.

These observations are in agreement with the hypothesis that IRAP translocation and shedding are strongly dependent upon insulin sensitivity.

## Conclusions

Type 2 diabetes and its preceding condition, prediabetes, are two major health issues worldwide, considering the dramatically increasing number of people having these conditions and the associated growing socioeconomic burden (Herman and Zimmet, 2012).

Interestingly and as opposed to other important health issues like cancer and infectious diseases, the major issue with T2D and PD is not so much their treatment, which is very cost-effective if started at an early stage and in constant progress with the advent of novel drugs, but timely diagnosis. The use of glycemia to screen for T2D and even more so for PD, has proven delusive probably due to its complex regulation and so are HbA1c and insulinemia.

IRAP, a protein associated with GLUT4 and directly involved in insulin-mediated glucose uptake, appears to be an interesting candidate biomarker for insulin-resistance.

We developed a highly specific and sensitive ELISA allowing the quantitave measurement of the circulating domain of IRAP in plasma and serum. The currently ongoing clinical trials will tell whether this test is a valuable tool for the screening and diagnosis of the insulin-resistance dependent diseases: prediabetes, type 2 diabetes and gestational diabetes.

## Data Availability Statement

The raw data supporting the conclusions of this article will be made available by the authors, without undue reservation.

## Ethics Statement

The studies involving human participants were reviewed and approved by Comité de protection des personnes Sud-Ouest et Outre-Mer III, NUMERO D'ENREGISTREMENT SICNRIPH:ID 2360 n°18.12.11.65552, NUMÉRO ID-RCB: 2018-A03158-47. The patients/participants provided their written informed consent to participate in this study.

## Author Contributions

CT, NG, AL, J-LC, CC, SL-P, ML, CG, KC, and SB participated to the studies. CG, HS, TB, FA-M, MB, and SB participated to the writing of the manuscript. MB and SB are the senior authors. All authors contributed to the article and approved the submitted version.

## Conflict of Interest

The authors declare that the research was conducted in the absence of any commercial or financial relationships that could be construed as a potential conflict of interest.
